# A profile of the mental health needs of children in out-of-home care in Wales between 2019-2020

**DOI:** 10.23889/ijpds.v9i5.2486

**Published:** 2024-09-18

**Authors:** Aimee Cummings

**Affiliations:** 1 Cardiff University

##  Objective and approach 

Monitoring the incidence of chronic health conditions (CHCs) in childhood in England using administrative data to derive numerators and denominators is challenged by unmeasured migration. We used a birth cohort design and different criteria for contact with health or education services to estimate the cumulative incidence of CHCs to age 16 years.

## Results

Mental health was considered in relation to two facets: indicators and conditions. Indicators included incidents of self-harm, drug or alcohol use, while conditions included symptoms or diagnoses of five different mental health conditions (anxiety, depression, eating disorder, conduct disorder, and severe mental illnesses). Prevalence was looked at across general demographic characteristics, as well as those specific to the care experience. Logistic regression models examined if a young person’s care experience could predict the likelihood of mental health need.

## Conclusions

The findings from this piece of work will inform current understanding of the vulnerabilities of this group. Future work will compare the needs of this group to others, including Children Receiving Care and Support, as well as the general population.

## Implications

This work will provide an update to existing literature. The findings have the potential to contribute to discussions about how we best support this group and will inform approaches to meeting the health needs of care-experienced young people.

